# Retraction Note: A Tunguska sized airburst destroyed Tall el-Hammam a Middle Bronze Age city in the Jordan Valley near the Dead Sea

**DOI:** 10.1038/s41598-025-99265-5

**Published:** 2025-04-24

**Authors:** Ted E. Bunch, Malcolm A. LeCompte, A. Victor Adedeji, James H. Wittke, T. David Burleigh, Robert E. Hermes, Charles Mooney, Dale Batchelor, Wendy S. Wolbach, Joel Kathan, Gunther Kletetschka, Mark C. L. Patterson, Edward C. Swindel, Timothy Witwer, George A. Howard, Siddhartha Mitra, Christopher R. Moore, Kurt Langworthy, James P. Kennett, Allen West, Phillip J. Silvia

**Affiliations:** 1 Geology Program, School of Earth and Sustainability, Northern Arizona University, Flagstaff, AZ 86011 USA; 2 Center of Excellence in Remote Sensing Education and Research, Elizabeth City State University, Elizabeth City, NC 27909 USA; 3 Department of Natural Sciences, Elizabeth City State University, Elizabeth City, NC 27909 USA; 4 Materials and Metallurgical Engineering, New Mexico Institute On Mining & Technology, Socorro, NM 87801 USA; 5 Los Alamos National Laboratory (Retired), Los Alamos, NM 87545 USA; 6 Analytical Instrumentation Facility, North Carolina State University, Raleigh, NC 27695 USA; 7 EAG Laboratories, Eurofins Materials Science, Raleigh, NC 27606 USA; 8 Department of Chemistry and Biochemistry, DePaul University, Chicago, IL 60614 USA; 9 Geophysical Institute, University of Alaska Fairbanks, 903 Koyukuk Drive, College, AK 99775 USA; 10 Faculty of Science, Charles University, Albertov 6, Prague, 12843 Czech Republic; 11 Southern Research Institute, 757 Tom Martin Drive, Birmingham, AL 35211 USA; 12 US Navy, NAVFAC Mid-Atlantic Region, NS Norfolk, VA 23511 USA; 13 Comet Research Group, Prescott, AZ 86301 USA; 14 Restoration Systems, L.L.C., Raleigh, NC 27604 USA; 15 Department of Geological Sciences, East Carolina University, Greenville, NC 27858 USA; 16 Savannah River Archaeological Research Program, South Carolina Institute of Archaeology and Anthropology, University of South Carolina, New Ellenton, SC 29809 USA; 17 CAMCOR, University of Oregon, 1443 E 13th Ave, Eugene, OR 97403 USA; 18 Department of Earth Science and Marine Science Institute, University of California, Santa Barbara, CA 93106 USA; 19 College of Archaeology, Trinity Southwest University, Albuquerque, NM 87109 USA

Retraction of: *Scientific Reports* 10.1038/s41598-021-97778-3, published online 20 September 2021

The Editors have retracted this Article.

Following publication of this Article concerns have been raised by Jaret & Harris^1^ about errors in methodology, analyses, and interpretation of the mineralogic and geochemical data in the Article. In addition, Boslough & Bruno^2^ show that comparisons between the Tall el-Hammam site and the Tunguska event are not adequately substantiated, as a result of errors propagated from the original sources that overestimated the temperature, wind speeds, and impact of the air blast at Tunguska.

Therefore, the claims that an airburst event destroyed the Middle Bronze Age city of Tall el-Hammam appear to not be sufficiently supported by the data in the Article. Given these concerns, the Editors no longer have confidence that the conclusions presented are reliable.

Malcolm A. LeCompte, T. David Burleigh, Robert E. Hermes, Wendy S. Wolbach, Gunther Kletetschka, Timothy Witwer, George A. Howard, Christopher R. Moore, James P. Kennett, Allen West, and Phillip J. Silvia disagree with the retraction. A. Victor Adedeji, James H. Wittke, Charles Mooney, Dale Batchelor, Joel Kathan, Mark C. L. Patterson, Edward C. Swindel, Siddhartha Mitra, and Kurt Langworthy did not respond to the Editors’ correspondence about the retraction. Ted E. Bunch is deceased.
